# Zoonoses in the workplace: A Seroprevalence study of *Coxiella*, *Brucella*, and *Leptospira* among marine mammal rescue and rehabilitation workers in California

**DOI:** 10.1002/puh2.132

**Published:** 2024-06

**Authors:** Adam Bjork, Robyn A. Stoddard, Alicia D. Anderson, Marie A. de Perio, Richard Todd Niemeier, Joshua S. Self, Kelly A. Fitzpatrick, Frances M. D. Gulland, Cara L. Field, Gilbert J. Kersh, John D. Gibbins

**Affiliations:** 1 Division of Vector-Borne Diseases, National Center for Emerging and Zoonotic Infectious Diseases (NCEZID), Centers for Disease Control and Prevention (CDC), Atlanta, Georgia, USA; 2 Epidemic Intelligence Service, CDC, Atlanta, Georgia, USA; 3 Division of High Consequence Pathogens and Pathology, NCEZID, CDC, Atlanta, Georgia, USA; 4 National Institute for Occupational Safety and Health, CDC, Cincinnati, Ohio, USA; 5 The Marine Mammal Center, Sausalito, California, USA

**Keywords:** biosafety, brucellosis, leptospirosis, marine mammal, Q fever, worker safety

## Abstract

**Background:**

Q fever, brucellosis, and leptospirosis are zoonoses typically associated with terrestrial animal reservoirs. These bacterial agents are now known to infect marine mammal species, though little is known about potential human health risks from marine mammal reservoir species. We investigated potential risks of these bacteria in humans associated with marine mammal exposure.

**Methods:**

The Marine Mammal Center (TMMC) in Sausalito, California, requested a Health Hazard Evaluation by the National Institute for Occupational Safety and Health. In June 2011, an investigation occurred, which included a written questionnaire and serosurvey among workers for *Coxiella burnetii, Brucella* spp., and Leptospira spp., and an environmental assessment for *C. burnetii*.

**Results:**

Serologic evidence of past exposure was detected in 4% (*C. burnetii*), 0% (*Brucella*), and 1% (*Leptospira*) of 213 participants, respectively. One of 130 environmental samples tested positive for *C. burnetii.* No significant marine mammal-specific risk factors were identified, but some safety deficiencies were noted that could lead to a higher risk of exposure to zoonotic diseases.

**Conclusion:**

Although this study did not identify disease exposure risks associated with marine mammals, additional studies in different settings of other groups with frequent exposure to marine mammals are warranted. Some deficiencies in safety were noted, and based on these, TMMC modified protocols to improve safety.

## INTRODUCTION

*Coxiella burnetii*, *Brucella*, and *Leptospira* spp. are zoonotic bacteria that cause Q fever, brucellosis, and leptospirosis, respectively. Most reported cases in humans appear to be due to transmission from terrestrial animals, but these bacteria infect marine mammals, and marine *Brucella* spp. have caused human disease [1–3]. Little is known about the potential human health risk of these pathogens associated with marine mammal reservoir species.

*C. burnetii* is distributed globally, is highly infectious, and can withstand harsh environmental conditions [4]. The most commonly recognized forms of acute Q fever in humans are a nonspecific flu-like illness, pneumonia, and hepatitis [4]. Transmission to humans occurs primarily via inhalation of aerosols or dust contaminated by animal excretions and birth products. Cattle, sheep, and goats are considered the primary *C. burnetii* reservoirs [5]. In recent years, *C. burnetii* infection has been detected in the placentas of the Pacific harbor seal, the Steller sea lion, the harbor porpoise, and the Northern fur seal [6–10]. The significance of these findings in relation to human Q fever infection is poorly understood. A human with Q fever endocarditis was reported in Greenland with harbor or hooded seals implicated as a possible source of infection [11].

Human brucellosis is found worldwide [12, 13]. Reservoirs for the *Brucella* spp. most commonly associated with human brucellosis include cattle, sheep and goats, pigs, and dogs [12, 13]. Humans are infected by contact with fluids from infected animals, such as ingestion of contaminated products (e.g., unpasteurized milk or cheese), contact with broken skin, or inhalation of contaminated aerosols [13]. Acute symptoms of human brucellosis most often include a nonspecific flu-like illness [13]. *Brucella* spp., specific to marine mammals, infect a variety of species, including pinnipeds, cetaceans, and otters [14]. To date, molecular analyses have linked marine mammal *Brucella* spp. to three naturally acquired, severe human illnesses and one laboratory-acquired illness [1–3].

Leptospirosis, caused by *Leptospira* spp., can also present in humans as an acute, nonspecific flu-like illness [15, 16]. Infection may result from direct contact with animal urine or from exposure to contaminated soil or water [15, 16]. Leptospirosis is a recreational hazard (e.g., swimming, wading, and paddling) and an occupational hazard (e.g., slaughterhouse workers, veterinarians, farmers, and sewer workers) [15, 16]. The only known marine mammal reservoir is the California sea lion; however, other species can be infected, including fur seals, elephant seals, and harbor seals [17–21]. Repeated outbreaks of leptospirosis in marine wildlife have occurred from southern California to British Columbia, killing large numbers of California sea lions [21]. *Leptospira* excreted by sea lions is also detectable in the sand along shorelines, indicating another potential route of transmission to humans [22].

The pathogenicity of marine mammal strains of these bacteria versus previously studied terrestrial strains is not known. Given their closer proximity to and increased likelihood of contact with marine mammals compared to the general population, marine mammal rescue and rehabilitation workers serve as an ideal population with which to investigate the potential transmission of these pathogens from marine mammals to humans. The prevalence of exposure to *C. burnetii*, *Brucella* spp., and *Leptospira* spp. among marine mammal workers is unknown. This health hazard evaluation assessed the occupational risk among workers at a large rehabilitation facility.

## METHODS

### Study design and setting

We conducted a cross-sectional study of occupational risk and evidence of exposure to *C. burnetii*, *Brucella*, and *Leptospira* among workers at The Marine Mammal Center (TMMC) in Sausalito, CA. TMMC is a nonprofit veterinary hospital and educational center focused on the rescue, treatment, and rehabilitation of marine mammals suffering from illness or injury. TMMC requested a health hazard evaluation by the National Institute for Occupational Safety and Health (NIOSH) because of concerns about the potential exposure of employees and volunteers to zoonotic diseases. At the time of the investigation, as many as 200 animals at a time (and approximately 1000 each year) were cared for at its facilities. The center routinely rehabilitates California sea lions, harbor seals, and elephant seals and sometimes has other marine species. Some staff members participate in whale necropsies in the field. The center’s workforce of approximately 600 volunteers and 35 paid employees at the time, respond to stranded marine mammals throughout 600 mi of the central and northern Pacific Coast of California. Facilities include the center’s headquarters (Sausalito, CA) and several field offices (Morro Bay and Moss Landing, CA), from which rescue operations are staged.

### Data and sample collection

Data and sample collection occurred in June 27–30, 2011. All employees and volunteers ≥18 years old who worked at the headquarters facility and triage centers on the dates of our evaluation were invited to participate in our convenience-sampled cross-sectional study. Other interested volunteers not working at the time were also welcome. All participants completed a written questionnaire that covered demographics, medical history, work duties, and potential exposures associated with Q fever, brucellosis, and leptospirosis (Supporting Information 1). Sera specimens were collected from participants upon completion of the questionnaire.

Environmental samples for *Coxiella* were collected by bulk, vacuum, swab, and sponge methods to assess the surface contamination of *Coxiella* at the Sausalito and Moss Landing locations. Bulk samples were taken by collecting 10–30 g of soil or debris into 50 mL tubes. Swab and sponge samples were collected by wiping the swab or sponge across a surface to collect dust or dirt from the surfaces. Samples were collected from various areas, such as offices, animal pens and transport cages, laboratories, walkways, common rooms, vehicles, and equipment. Vacuum air samples were collected from set locations (i.e., area samples) or from a vacuum pump and filter attached to a worker while performing job duties (i.e., a personal breathing zone). Personal breathing zone and area air samples were collected on 37-mm diameter, 0.3-*μ*m pore size polytetrafluoroethylene filters. The filter cassettes were connected to battery-powered SKC AirChek 2000 sampling pumps (SKC Incorporated, Eighty Four, Pennsylvania) and operated at 3 L/min. All pumps were pre- and post-calibrated with the sampling media connected. The collection and extraction efficiencies of this filter for another bacterial agent have been previously described by Burton et al. [23, 24]. Field blanks were collected at a rate of 10% of actual samples collected.

### Laboratory testing

For Q fever testing, the specimens were screened by an enzyme-linked immunosorbent assay (ELISA) for IgG antibodies against phase II *C. burnetii* [25]. If positive or equivocal, they were tested by indirect immunofluorescent antibody assay to confirm the ELISA results and to determine IgG and IgM titers against phases I and II *C. burnetii* antigens [25]. Serologic evidence of past exposure to Q fever was defined as a phase I or II IgG titer ≥1:32. A phase I IgG titer of ≥1:800 was defined as suggestive of chronic Q fever, though the confirmation of chronic Q fever requires additional clinical assessments that were not part of this evaluation [26]. For brucellosis testing, sera were tested by the *Brucella* microagglutination test (MAT), as previously described with minor modifications, including use of U-bottom plates, incubation at 27°C, and discontinued use of safranin [27]. Serologic evidence of past exposure to *Brucella* was defined as an IgG titer of ≥1:160 [28]. The *Leptospira* MAT was used for leptospirosis testing, with an IgG titer of ≥1:100 as the cutoff level used to define serologic evidence of past exposure [29, 30]. Twenty live leptospiral cell suspensions were tested (Table 1). Antigens were incubated with serially diluted serum specimens, and resulting agglutination titers were read using darkfield microscopy. A titer below these cutoff levels was considered too low to determine if a participant was previously infected, due to the potential for cross-reactivity with other pathogens and the unknown timing of exposure or clinical illness over the participant’s lifetime.

Environmental samples were tested for evidence of *C. burnetii* by quantitative PCR (qPCR), which estimated the number of genome equivalents present in a sample using an outer membrane protein-coding gene, COM1, and a multi-copy insertion element, IS1111 [31, 32]. Environmental samples were not tested for *Brucella* or *Leptospira*.

### Data analysis

Prevalence ratios were calculated, and statistical differences in exposure histories and characteristics (between seropositive and non-seropositive workers) were determined using the Fisher exact test. To determine if the duration of time working at TMMC was significant for seropositive versus seronegative workers, a two-tailed *t*-test was done. Statistical analyses were performed using EpiInfo version 3.5.3 and Microsoft Excel. The data collected for the study were stored on secure CDC servers.

### Ethical considerations

Each participant was informed in writing of his or her individual test results and their significance. As a public health response, per the guidelines of Title 45 Code of Federal Regulations Part 46, this evaluation was determined to not require review by an institutional review board [33].

## RESULTS

A total of 222 participants completed the questionnaire, including 35 paid employees, 184 volunteers, and 3 participants who reported working both as a paid employee and a volunteer. Among the 222 participants, 198 (89%) worked at the headquarters, 24 (11%) worked at the triage facility, and 213 (96%) reported caring for or handling marine mammals. The median age of participants was 45 years (range: 18–77 years), and 164 (74%) were female. Survey participants had worked/volunteered at the headquarters or triage facility for a median of 2.4 years (range: 2 days to 35 years), 40 weeks/year (range: 1–52 weeks/year), and 8 h/week (range: 2–70 h/week). Eleven participants (5%) reported being veterinarians, and 40 (18%) described themselves as working full-time for the facility.

A total of 213 (96%) of the 222 participants who completed the survey provided a serum sample for *Coxiella*, *Brucella*, and *Leptospira* testing. Evidence of past exposure to *C. burnetii* was detected in 9 (4%) of these 213 participants, with phase I IgG titers of 1:32 (1 individual), 1:64 (4 individuals), and 1:128 (4 individuals). None had titers suggestive of chronic Q fever infection. One participant, a veterinarian, reported a previous diagnosis with Q fever in 2004, before working at the facility. The remaining eight were identified through this investigation.

Median age of the nine exposed individuals was 40 years (range, 27–60 years). Eight (89%) were female, three (33%) were paid employees, six were volunteers, and eight (89%) reported caring for or handling marine mammals. Duration of time working at TMMC was not significantly associated with seropositivity. Characteristics significantly associated with seropositivity (Table 2) included being a veterinarian (prevalence ratio [PR] = 11.6, *p* < 0.01), self-reported exposure to feral swine blood or fluids in past year (PR = 11.9; *p* = 0.01), and consumption of raw (unpasteurized) dairy products such as raw milk or cheese within the last year (PR = 4.4; *p* = 0.03). A history of miscarriage was reported more commonly among seropositive women (*n* = 3/8) than non-seropositive women (*n* = 14/149) (PR = 4.9; *p* = 0.04). None of the 17 females reporting a miscarriage were veterinarians. No other reproductive outcomes, including history of stillbirth, premature delivery, or low birth weight baby were significantly associated with *C. burnetii* seropositivity.

Evidence of previous exposure to *Leptospira* was detected in two (1%) part-time volunteers at the center aged 20–25 years. Sample sizes were too small to detect significant exposure risks, if any. One reported caring for or handling marine mammals and had a titer of 1:200 for *Leptospira interrogans* serovar Icterohaemorrhagiae; the other had a titer of 1:200 for *L. interrogans* serovar Cynopteri, though positive antibodies for these serovars do not indicate definitively that these were the infecting serovars [30, 34, 35]. No participants showed serologic evidence of previous infection with both *C. burnetii* and *Leptospira*. No participants were found to have serologic evidence of previous exposure to *Brucella*. Thirty-eight (18%) participants had positive *Brucella* antibody titers less than the cutoff titer of 1:160. Although this could indicate past infection with *Brucella* spp., it could also be the result of cross-reactivity on *Brucella* serological tests caused by previous infection with other common gram-negative bacteria. Only titers above the determined threshold were considered evidence of past infection in this investigation.

A total of 130 environmental samples were collected during the investigation (49 sponge/swab, 12 bulk, 25 vacuum, and 44 air samples [21 area samples, 23 personal breathing zone samples]). Eighteen (14%) environmental samples were collected from a field office, and 112 (86%) were collected at the headquarters. Only one sample—from a room used to prepare fish food for the rescued animals—tested weakly positive for *C. burnetii* by IS1111 qPCR (approximately 38.4 genome equivalents) but negative by COM1 qPCR. The IS1111 target is a multi-copy gene and therefore makes the assay more sensitive, but some marine mammal isolates do not have the IS1111 target sequence. Therefore, the less sensitive single-copy *com1* target was also used to ensure inclusion of all marine mammal strains.

During the evaluation of safety practices, there were some observations of work practices that could lead to a higher risk of exposure to zoonotic potential (Table 3). Highlights of findings included: (1) some employees and volunteers were not wearing the correct personal protective equipment (PPE); (2) the biological safety cabinet in the laboratory did not have enough airflow; and (3) the ventilation system in the harbor seal area blew air from the intensive care unit to the other areas of the building.

## DISCUSSION

Among this group of marine mammal workers in California, *C. burnetii* seroprevalence was 4%, a figure comparable to the 3% seroprevalence estimated for the general US adult population [25]. Though no seroprevalence estimates for the general US population are available for *Leptospira* or *Brucella*, we found low seroprevalences of 1% (2 participants) and 0% (0 participants) for these bacteria, respectively.

None of the risk factors associated with prior exposure to *C. burnetii* (i.e., ingestion of unpasteurized dairy products; contact with feral swine blood or fluids; and a veterinary occupation) implicated marine mammals or work related to marine mammal rescue or rehabilitation [36]. These significant risk factors may suggest that detected titers were due to past exposure to other animals besides marine mammals. Studies of US veterinarians estimate a 22% seroprevalence for *C. burnetii* and a 0%–3% seroprevalence for *Leptospira* [37, 38]. During an outbreak of leptospirosis in canines, there was no evidence of zoonotic transmission to humans with high-risk exposure [39]. In the present study, 36% (4 of 11; 95% CI [11%, 69%]) of participants identified as veterinarians were *C. burnetii* seropositive, and 0% (0 of 11; 95% CI [0%, 11%]) were *Leptospira* seropositive.

An overrepresentation of miscarriages was detected among serosurvey respondents who were *C. burnetii* seropositive versus non-seropositive females. However, all three seropositive participants reporting a miscarriage experienced their miscarriages prior to working at the center. Furthermore, it is not known if these miscarriages were the result of prior Q fever infections. Although there does not appear to be a connection between marine mammal contact and Q fever, adverse outcomes are known to be associated with as many as 80% of pregnant women infected with *C. burnetii* during pregnancy [40].

The zoonotic and pathogenic potential for humans of the strains of *C. burnetii*, *Leptospira*, and *Brucella* in marine mammal reservoirs is not well understood. In this study, marine mammal workers did not appear to have a higher incidence of *C. burnetii* seropositivity than the general US population, despite evidence of *C. burnetii* infection in many marine mammal species. Prevalence of *C. burnetii* infection among marine mammals treated and handled by the study population of marine mammal workers is not known. The *C. burnetii* strains that infect marine mammals have distinct genetic markers compared to strains from terrestrial species, and the virulence of the marine mammal strains in humans is not known [10, 41]. It is therefore possible that human infection could be low, even if high percentages of the animals were infected with *C. burnetii*. Further studies could evaluate the potential for marine mammal *C. burnetii* isolates to infect humans and other mammals.

The environmental sampling in this site also did not reveal widespread contamination with *C. burnetii*. This was unexpected, as *C. burnetii* can commonly be found in many environments and may indicate a low prevalence of infection among the animal population handled and cared for at the rescue center. A previous study detected the bacteria in nearly 25% of surface samples collected not only from dairy farms and ranches but also from many facilities where animals are not normally encountered [41]. The failure to detect higher environmental loads of *C. burnetii* at the facilities examined or to find high seroprevalences of any of the diseases under investigation suggests that either [1] the infections are uncommon in the marine mammal population served by the facilities or [2] the potential for contamination is limited at both facilities. The first possibility is unlikely based on the data available for *C. burnetii*. For instance, *C. burnetii* seropositivity was detected in 34% of 215 Pacific harbor seals sampled in the Pacific Northwest, though, as noted above, the seroprevalence of rescued marine mammal populations may differ depending on their age and reproductive status [7]. The second possibility is more likely: *C. burnetii* infection is most commonly associated with dry and dusty environments, as these conditions are amenable to the bacterium becoming airborne and spreading to areas away from the shedding animal. The marine environment is neither dry nor dusty, and the facilities examined in this investigation were observed to be regularly sprayed with water with disinfectant and cleaned. This may limit the potential for the bacterium to be transmitted to humans and surfaces away from infected animals.

There are some limitations to the serosurvey and questionnaire. First, the presence of antibodies in a single serum specimen indicates only that a participant has been exposed to or infected with the agent in question at some point in the past. It is not possible to determine when or from what reservoir species that infection occurred. Second, the majority of participants were volunteers at the facility’s headquarters. The exposure risks and histories of these workers may differ from responders working in less controlled and hygienic environments (e.g., on shores or in transport vehicles) with closer exposure to animals that have not been clinically evaluated. Third, given that exposure history was determined using questionnaires, recall bias may be present in the data. Fourth, background seroprevalence estimates are not available for comparison across the region where the investigation took place. Finally, we only did environmental sampling for *Coxiella*; time constraints and the challenges of testing for *Leptospira* and *Brucella* spp. prevented their inclusion in the environmental portion of our study.

The results of this investigation suggest a low occupational risk of Q fever, leptospirosis, or brucellosis among this group of marine mammal workers on the Pacific Coast of California. Nevertheless, appropriate safety precautions ensure maximal protection while working with marine mammals. Pregnant women, immunosuppressed persons, and patients with pre-existing heart valve defects, arterial aneurysms, or vascular grafts are advised to limit their exposure to marine mammals to reduce the risk of infection with the zoonotic pathogens studied here and other pathogens associated with marine mammals. The workers participating in the present study were advised to wash their hands regularly and to keep work clothing, including rubber boots worn in animal pens, out of designated “clean” areas (e.g., meeting rooms and dining areas). They were also advised to use face shields and N95 filtering facepiece respirators or higher during animal surgeries and necropsies, and to include personnel performing these activities in the facility’s respiratory program, in accordance with national guidelines and regulations [42, 43].

As a result of this investigation, additional measures to protect marine mammal workers from zoonotic disease exposure have been established at TMMC. A Health and Safety Committee helps identify and mitigate risks. Employees and volunteers are now given additional educational training on zoonotic disease risks and the importance of PPE in preventing disease transmission. Higher risk individuals receive specific information about prevention. Animal care clothing and boots are not permitted in dining and public areas, and frequent hand washing is emphasized. Structural and functional equipment adjustments have improved airflow and ventilation in areas that were identified as potentially higher risk, as well as changes in the management of animals undergoing intensive care. Animals are transported in vehicles with complete separation of airflow. Prior to the COVID-19 pandemic, the use of face shields and N95 respirators in higher risk situations remained inconsistent. However, the pandemic greatly emphasized risks associated with respiratory and other pathogens, resulting in a higher level of compliance with respiratory and other PPE than at any other time.

## CONCLUSION

We found low serologic evidence of past exposure to *C. burnetii* and *Leptospira*, and no evidence of past exposure to *Brucella*, among TMMC workers. This raises additional questions for future studies to determine if the results from TMMC are reflective of other high-risk populations. The potential for exposure and infection may vary from group to group. This can be investigated through additional studies focusing on other groups with high levels of exposure to marine mammals, such as marine biologists and native populations that hunt marine mammals and are less likely to be wearing appropriate PPE. Studies could also include the sampling of animals at the same time as the humans exposed to them to determine if the animals are infected.

## Supplementary Material

Questionnaire

## Figures and Tables

**TABLE 1:** Panel of *Leptospira* species, serogroup, serovar, and strain live antigens used for the microagglutination test.

Species	Serogroup	Serovar	Strain
*L. interrogans*	Australis	Australis	Ballico
*L. interrogans*	Australis	Bratislava	Jez-Bratislava
*L. interrogans*	Autumnalis	Autumnalis	Akiyama A
*L. borgpetersenii*	Ballum	Ballum	Mus 127
*L. interrogans*	Bataviae	Bataviae	Van Tienen
*L. interrogans*	Canicola	Canicola	Ruebush
*L. weilii*	Celledoni	Celledoni	Celledoni
*L. kirschneri*	Cynopteri	Cynopteri	3522 C
*L. interrogans*	Djasiman	Djasiman	Djasiman
*L. kirschneri*	Grippotyphosa	Grippotyphosa	Moskva V
*L. santarosai*	Hebdomadis	Borincana	HS 622
*L. interrogans*	Icterohaemorrhagiae	Icterohaemorrhagiae	RGA
*L. interrogans*	Icterohaemorrhagiae	Mankarso	Mankarso
*L. borgpetersenii*	Javanica	Javanica	Veldrat Bataviae 46
*L. santarosai*	Mini	Georgia	LT 117
*L. interrogans*	Pomona	Pomona	Pomona
*L. santarosai*	Pyrogenes	Alexi	HS 616
*L. interrogans*	Pyrogenes	Pyrogenes	Salinem
*L. interrogans*	Sejroe	Wolfii	3705
*L. borgpetersenii*	Tarrasovi	Tarrasovi	Perepelitsin

**TABLE 2:** Frequency and prevalence of *Coxiella burnetii* seropositivity in participants from The Marine Mammal Center, Sausalito, CA, June 2011.

	No. infected/total^a^ (% infected)			
Characteristic or exposure	Participants with characteristic or exposure	Participants without characteristic or exposure	Prevalence ratio	*p*-Value
Sex (female)	8/157 (5)	1/56 (2)	2.9	0.45
**Veterinarian**	**4/11** (36)	**5/159** (3)	**11.6**	**<0.01**
Cared for or handled				
Pinnipeds (seals, fur seals, sea lions)	8/204 (4)	1/9 (11)	0.4	0.33
Cetaceans (whales, dolphins, porpoises)	4/78 (5)	4/133 (3)	1.7	0.47
Sea otters	3/58 (5)	6/154 (4)	1.3	0.71
Contact with				
Live marine mammal (including tissue, fluid, blood)	9/200 (5)	0/13 (0)	Undefined	>0.99
Dead marine mammal (including tissue, fluid, blood)	7/162 (4)	2/51 (4)	1.1	>0.99
Pregnant/newborn marine mammal, or marine mammal birth products	7/132 (5)	2/81 (2)	2.1	0.49
Clean or repair equipment or enclosures	7/197 (4)	2/16 (13)	0.3	0.14
Ever lived within 5 mi of sheep, goat, cattle	5/84 (6)	4/129 (3)	1.9	0.32
**Exposed to feral swine blood/fluids in past year**	**2/5** (40)	**7/208** (3)	**11.9**	**0.01**
**Consumed raw dairy in past year**	**5/47** (10)	**4/166** (2)	**4.4**	**0.03**

*Note:* Characteristics with *p*-values (Fisher exact test) <0.05 shown in bold text.

a Total respondents who provided a serum sample for *Coxiella* testing was 213, although blank responses to some questions reduced the denominator for some fields.

**TABLE 3:** Frequency of work practices reported by employees and volunteers who also reported caring for marine mammals, The Marine Mammal Center, Sausalito, CA, June, 2011.

	No. (%) participants, *n* = 211–213^a^			
Work practice	Always	Most of the time	Some of the time	Never
Wash hands before eating	194 (91)	17 (8)	2 (1)	0 (0)
Change clothes before eating	63 (30)	37 (17)	53 (25)	59 (28)
Shower before eating	7 (3)	10 (5)	38 (18)	158 (74)
Change clothes before leaving work	61 (29)	17 (8)	55 (26)	80 (38)
Shower before leaving work	2 (1)	5 (2)	16 (8)	190 (89)
PPE use				
Gloves	154 (73)	47 (22)	10 (5)	0 (0)
Rubber boots	146 (69)	41 (19)	23 (11)	3 (1)
Rain coat/waterproof suit	88 (41)	58 (27)	52 (24)	15 (7)

Abbreviation: PPE, personal protective equipment.

a Sample sizes vary because of missing values.

## Data Availability

The data that support the findings of this study are available on request from the corresponding author. The data are not publicly available due to privacy or ethical reasons.
