# Supplementary material for: Zoonoses in the workplace: A Seroprevalence study of Coxiella, Brucella, and Leptospira among marine mammal rescue and rehabilitation workers in California
Source: Public Health Chall. 2024 Jun 27;3(2):e132. doi: 10.1002/puh2.132 (PMC11951298; doi:10.1002/puh2.132)
Supplement: Supplementary file 1 — Supporting Information 1 Questionnaire used by the National Institute for Occupational Safety and Health during a health hazard evaluation at The Marine Mammal Center, Sausalito, CA, June 2011. [file PUH2-3-e132-s001.docx]

Form Approved

0920-0260

Expires 1/31/12

**U.S. Department of Health and Human Services**

**U.S. Public Health Service**

**Centers for Disease Control and Prevention**

**National Institute for Occupational Safety and Health**

**The Marine Mammal Center Questionnaire**

**Sausalito, CA**

**June 2011**

This questionnaire is part of a National Institute for Occupational Safety and Health (NIOSH) evaluation to examine the prevalence of Q fever, brucellosis, and leptospirosis among employees and volunteers at The Marine Mammal Center. This questionnaire includes questions about your personal characteristics, work history, and relevant medical history.

Participation in this survey is voluntary – there is no penalty for choosing not to participate. However, full participation will better enable NIOSH to recommend strategies for reducing your exposure to the above infections. Please answer all questions to the best of your ability.

All personal information from this questionnaire will be kept confidential according to federal law. Group summary results of this evaluation (without any personal identifying information) will be provided to the management and employees of The Marine Mammal Center in the form of a final report that will be prepared after the survey is complete.

Public reporting burden for this collection of information is estimated to average 20 minutes per response, including the time for reviewing instructions, searching existing data sources, gathering and maintaining the data needed, and completing and reviewing the collection of information. An agency may not conduct or sponsor, and a person is not required to respond to, a collection of information unless it displays a currently valid OMB control number. Send comments regarding this burden estimate or any other aspect of this collection of information including suggestions for reducing this burden to: CDC, Project Clearance Officer, 1600 Clifton Road, MS D-24, Atlanta, GA 30333, ATTN: P.A. (0920-0260). Do not send the completed form to this address.

*Section to be completed by CDC/NIOSH investigators:*

Date of Questionnaire: _____/_____/ 2011

Questionnaire reviewed by: Gibbins dePerio Anderson Bjork

**Section I: Personal Characteristics**

1) What is your age? ______ years

2) What is your sex:  Male  Female

3) Which one of these groups would you say best represents your race? Please choose one.

 American Indian or Alaskan Native

 Asian

 Black or African American

 Native Hawaiian or other Pacific Islander

 White

 Other, please specify:____________________

4) What is your ethnicity?

 Hispanic or Latino

 Not Hispanic or Latino

**Section II: Work History**

5) Are you currently a paid employee or volunteer at The Marine Mammal Center (TMMC)?

 Paid employee

 Volunteer

6) Do you currently work/volunteer full-time or part-time at TMMC?

 Full-time

 Part-time

7) Since you began at TMMC, on average:

7a) How many weeks a year have you typically worked/volunteered? ______weeks/year

7b) How many hours per week have you typically worked/volunteered? ______hours/week

8) How long have you worked/volunteered at TMMC? ______ years ______ months

9) ***If you are a paid employee*,** what is your current job title at TMMC?

__________________________________

10) ***If you are a volunteer****,* please provide the following information for your paid job. If you do not have a paid job please check does not apply

□ Does not apply

Occupation: ______________

Name of workplace: _______________

City:________________

State:_______________

11) ***If you are a volunteer****,* please check the areas in which you volunteer *(check all that apply)*

□ Animal care-Topside

□ Animal care-Harbor Seals

□ Stranding

□ Education/Docent

□ Other: (*please specify*)____________________________

12) Have you ever cared for/handled the following types of marine mammals at TMMC? (*If yes, indicate the number of days per year)*

|  | **Yes** | **No** | **If yes, indicate number**  **of days/year** |
| --- | --- | --- | --- |
| Sea lions | □ | □ | _____ days/year |
| Seals | □ | □ | _____days/year |
| Dolphins | □ | □ | _____days/year |
| Porpoises | □ | □ | _____days/year |
| Whales | □ | □ | _____days/year |
| Sea otters | □ | □ | _____days/year |
| Fur seals | □ | □ | _____days/year |

13) Since working/volunteering at TMMC, have you ever had the following exposures *(Check all that apply.)*

□ Direct contact with live marine mammals while you are in the water

□ Direct contact with live marine mammals while you are out of water

□ Contact with tissue or blood from a live marine mammal

□ Contact with marine mammal body fluids (feces, urine, vomit)

□ Cleaning or repairing enclosures/equipment used for marine mammals

□ Contact with dead marine mammals not involving necropsy activities

□ Contact with dead marine mammal tissues, fluids, and blood during necropsy

□ Other (*please specify*)

________________________________________________________________________ _________________________________________________________________________

14) When working/volunteering for TMMC, have you ever done the following activities? (*If yes, indicate the number of days per year)*

|  | **Yes** | **No** | **If yes, indicate number of days/ year** |
| --- | --- | --- | --- |
| Care for pregnant marine mammals | □ | □ | □ |
| Care for newborn marine mammals (<1 wk old) | □ | □ | □ |
| Assist with/present at birthing | □ | □ | □ |
| Have contact with birth products (placenta, amniotic fluid, blood, soiled bedding) | □ | □ | □ |

15) When working/volunteering for TMMC, how often have you done the following?

|  | **Always** | **Most of the time** | **Some of the time** | **Never** |
| --- | --- | --- | --- | --- |
| Wear protective eye goggles | □ | □ | □ | □ |
| Wear mask | □ | □ | □ | □ |
| Wear respirator | □ | □ | □ | □ |
| Wear lab coat | □ | □ | □ | □ |
| Wear rain coat/waterproof suit | □ | □ | □ | □ |
| Wear gloves | □ | □ | □ | □ |
| Wear rubber boots | □ | □ | □ | □ |
| Wash hands before eating | □ | □ | □ | □ |
| Change clothes before eating | □ | □ | □ | □ |
| Shower before eating | □ | □ | □ | □ |
| Change clothes before leaving work | □ | □ | □ | □ |
| Shower before leaving work | □ | □ | □ | □ |

16) When working/volunteering for TMMC, at which locations have you handled marine mammals? (*Check all that apply)*

□ TMMC facilities

□ Rookery in California

□ Hawaiian Islands

□ Alaska

□ Other: (please specify) _______________________________

**Section III: General Exposures**

17) Have you ever lived on or within 5 miles of a sheep, goat, or cattle property?

□ Yes *(please answer 17a below)*

□ No

17a) Which species *(circle all that apply)* sheep goat cattle

___________Number of years

18) Had you ever worked with animals prior to TMMC?

□ Yes *(If yes, please answer 18a below)*

□ No

18a) If you answered **yes**, please list which species and period of time you worked with that

species

| **Species** | **Years worked with that species** |
| --- | --- |
|  | years |
|  | years |
|  | years |
|  | years |
|  | years |

19) Since you began working/volunteering at TMMC, have you worked with animals outside of your TMMC activities?

□ Yes *(If yes, please answer 19a below)*

□ No

19a) If you answered **yes**, please list which species and period of time you worked with that

species

| **Species** | **Years worked with that species** |
| --- | --- |
|  | years |
|  | years |
|  | years |
|  | years |
|  | years |

20) Have you ever participated in outdoor water recreational activities (e.g. water sports, swimming, paddling)? Please exclude activities involving swimming pools.

□ Yes

□ No

21) **Within the last year**, have you been exposed to blood or other body fluids from feral (wild) swine?

□ Yes □ No

22) **Within the last year**, have you eaten raw (unpasteurized) dairy products, such as raw milk or raw cheeses?

□ Yes

□ No

**Section IV: Medical History**

*The diseases that we are testing for may rarely cause medical problems such as heart disease, liver disease or reproductive problems. The following questions are related to potential complications of these diseases.*

23) Have you ever been diagnosed with any of the following? *(If yes, indicate method and year of diagnosis if known)*

|  | **Yes** | **No** | **If yes, indicate method and year of diagnosis** |
| --- | --- | --- | --- |
| Q fever | □ | □ |  |
| Brucellosis | □ | □ |  |
| Leptospirosis | □ | □ |  |

24) Have you ever been told by a doctor that your immune system is suppressed or compromised, or have you taken medications that impair your immune system (e.g. steroids, immunosuppressive drugs, or chemotherapy)?

□ Yes

□ No

25) Have you ever contracted a disease from animals?

□ Yes

□ No

25a) If you answered **yes**, please explain: __________________________________**____________________________________________________________________________________________________________________________________________________________________________________________**

26) Have you ever had any of the following conditions or procedures or been treated for any of the following diseases? *(Please check all that apply)*

| **Check if yes** | **Condition** |
| --- | --- |
| **□** | Arthritis or arthritis-like disease |
| **□** | Chronic back pain |
| **□** | Chronic joint problems |
| **□** | Endocarditis (inflammation of heart lining) |
| **□** | Artificial heart valve |
| **□** | Heart murmur or valve disease |
| **□** | Vascular graft (heart by-pass surgery is an example) |
| **□** | Pneumonia |
| **□** | Liver disease or hepatitis |
| **□** | Meningitis |
| **□** | Kidney disease |
| **□** | ***(For men only):*** Orchitis/epididymitis (inflammation/swelling of the testes/epididymis) |
| **□** | Pulmonary hemorrhage (bleeding from the lung) |
| **□** | Uveitis (inflammation of the eye: iris, choroid, or ciliary body) |
| **□** | Pancreatitis |
| **□** | Abnormal fatigue or tiredness |
| **□** | Insomnia/difficulty sleeping |
| **□** | Night sweats |

26a) If you answered **yes** to any of the above questions #23-26, please explain:

_________________________________________________________

_________________________________________________________

_________________________________________________________

26b) If you answered **yes to any of the above questions #23-26,** will you give NIOSH

permission to obtain your medical records at no cost to you? □ Yes □ No

(If yes, reviewer will give you an authorization form to sign)

27) ***[For women only]*** Have you **ever** experienced any of the following?

|  | **Yes** | **No** |
| --- | --- | --- |
| Miscarriage | □ | □ |
| Stillbirth | □ | □ |
| Low birth weight baby | □ | □ |
| Premature baby | □ | □ |

27a) If you answered **yes**, please explain: ____________________________

**______________________________________________________**

**Thank you for your participation.**
